# When hiccups mean something more than just a nuisance: A rare presentation of pyogenic liver abscess

**DOI:** 10.1002/jgh3.12591

**Published:** 2021-06-22

**Authors:** Muaaz Masood, Pearl Uy, John E Yap

**Affiliations:** ^1^ Department of Internal Medicine Medical College of Georgia at Augusta University Augusta Georgia USA; ^2^ Department of Gastroenterology and Hepatology Medical College of Georgia at Augusta University Augusta Georgia USA

**Keywords:** gastroenterology, gastroenterology infections, hepatology, hepatic inflammation

## Abstract

Pyogenic liver abscess (PLA) is an uncommon yet potentially fatal disease. The disease most commonly arises from biliary infection but may also result from hematogenous seeding and portal spread secondary to bowel contamination, direct seeding, or penetrating trauma. The diagnosis is suspected when there is a hepatic lesion on imaging. Confirmation of diagnosis requires purulent aspirate or bacterial growth on Gram stain/culture of the abscess or blood. The mainstay of treatment is antimicrobials in conjunction with either percutaneous abscess drainage or aspiration. Surgical drainage is reserved for cases of ruptured abscess, peritonitis, or in the presence of an underlying surgical cause. PLA typically presents with fever and abdominal symptoms. We report a case of an unusual manifestation of PLA, presenting as hiccups, which led to a significant delay in the diagnosis and treatment.

## Graphical abstract

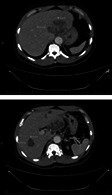

Pyogenic liver abscess (PLA) is an uncommon yet life‐threatening condition that typically presents with fever, jaundice, and abdominal pain. We report an unusual case of PLA manifesting as hiccups. This case highlights the importance of thoroughly investigating persistent or intractable hiccups for an underlying occult cause including the possibility of a PLA to prevent a delay in the diagnosis and treatment of this highly fatal disease.

## Introduction

Pyogenic liver abscess (PLA) is a life‐threatening condition that carries a mortality rate of 2–12%.^1^ The annual incidence of PLA has been reported to be 2.3 cases per 100 000.^2^ The disease has a slight predilection for men over women. Risk factors for PLA include diabetes mellitus, hepatic surgery, prior liver transplant, pancreatic disease, hepatobiliary disease including malignancy, biliary tract surgery, and the use of proton pump inhibitors.^3^ PLA commonly presents with fever, jaundice, and abdominal pain. We present an unusual case of PLA manifesting as hiccups, which led to a significant delay in the diagnosis and treatment.

## Case Report

**Figure 1:**
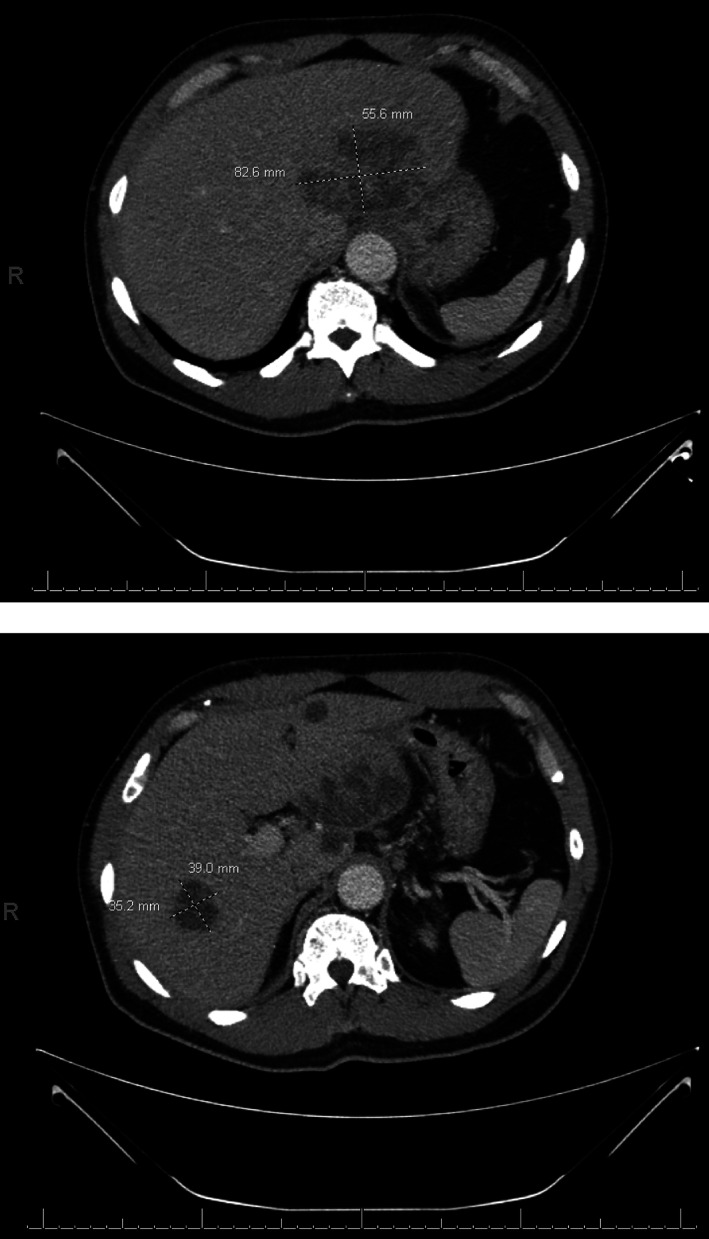
Axial views of contrasted abdominopelvic computed tomography scan demonstrating diffuse, multiloculated hypodense lesions consistent with liver abscesses, with the largest lesions measuring approximately 8.3 × 5.6 cm (top) and 3.9 × 3.5 cm (bottom) in size.

A 63‐year‐old African American male with a history of hypertension and anemia presented with intractable hiccups and fevers for 2 weeks. He denied dysphagia, shortness of breath, cough, abdominal pain, nausea, vomiting, jaundice, changes in stool, and dysuria. The patient initially visited an outside hospital 1 week prior to presentation for similar symptoms and was discovered to have acute kidney injury, pneumonia, and elevated liver enzymes. He was then admitted for 3 days, treated with fluids and intravenous antibiotics prior to being discharged home on oral antibiotics. He continued to experience hiccups, which prompted him to return to the same hospital's emergency department where he was prescribed oral diazepam and discharged home. Patient now sought a second opinion for persistent hiccups and ongoing fevers. Upon arrival, vital signs were notable for blood pressure 150/110 mmHg, heart rate 101 bpm, and temperature 38.9°C. Physical examination revealed an ill‐appearing, diaphoretic male with a soft, non‐tender, non‐distended abdomen. Laboratory studies were significant for leukocytosis with white blood cell count (WBC) of 23 400 mm^3^, segmented neutrophils 85%, hemoglobin 13.6 g/dL, mean corpuscular volume 84.2 fL, creatinine 1.12 mg/dL, aspartate aminotransferase 17 U/L, alanine aminotransferase 37 U/L, alkaline phosphatase 111 U/L, and total bilirubin 0.7 mg/dL. Contrast‐enhanced, abdominopelvic computed tomography (CT) scan revealed acute sigmoid diverticulitis and diffuse, multiloculated hypodense lesions consistent with liver abscesses, with the largest lesions measuring 8.3 × 5.6 cm and 3.9 × 3.5 cm in size (Fig. 1). Patient was started on intravenous piperacillin–tazobactam and underwent percutaneous abscess drainage. Fine needle aspiration yielded 5 mL of purulent aspirate. Bacterial culture did not show growth after 5 days. He was also started on baclofen. Patient's clinical condition subsequently improved with complete resolution of his hiccups, and he was discharged home on a 6‐week course of oral antibiotics ciprofloxacin and metronidazole.

## Discussion

Hiccups, also known as singultus, are a common occurrence caused by involuntary, repetitive myoclonic contractions of the diaphragm and intercostal muscles. Hiccups are classified according to their duration: acute (<48 h), persistent (>48 h), and intractable (>1 month). Acute hiccups tend to be self‐limited, transient, and typically do not warrant extensive work‐up. Persistent or intractable hiccups require further investigation for pathology involving the diaphragm, gastrointestinal, cardiopulmonary, or central nervous systems. Medication‐induced and psychogenic causes should also be explored. The most common causes of hiccups include gastroesophageal reflux disease, Parkinson's disease, anxiety, and gastric distension secondary to irritants, i.e. alcohol, tobacco. Serious diseases have been reported to present with hiccups such as pulmonary embolism, myocardial infarction, cerebrovascular accident, subdural hematoma, brain tumor, and intracranial aneurysm.^4^ Finally, medications including benzodiazepines, opioids, and dopamine agonists may also be associated with hiccups. A thorough history of presenting illness, pertinent physical examination, relevant laboratory data, and imaging studies will aid in narrowing the differential diagnoses. Persistent hiccups are typically a very bothersome and distressing symptom. Several medications have been used to provide symptomatic relief including baclofen, chlorpromazine, metoclopramide, gabapentin, and amitriptyline.^5^

Liver abscesses may pose as an irritant to the diaphragm and be a rare cause of persistent hiccups. There are only two published reports in the literature to our knowledge that describe hiccups as an initial presentation for a PLA.^6^, ^7^ In our case, the patient did not have any localizing abdominal signs or symptoms. We suspected that the PLA resulted from seeding of bacteria from the site of diverticulitis.

The diagnosis of PLA is established when there is a hepatic lesion on imaging (i.e. ultrasound and CT) in addition to purulent aspirate or bacterial growth on Gram stain/culture of abscess or blood. The mortality of PLA has declined in recent years likely due to improved imaging and diagnostic techniques.^8^ The mainstay of treatment is antimicrobials in conjunction with abscess drainage. Percutaneous drainage is the preferred approach and has been reported to have a similar or decreased mortality rate when compared with open surgical drainage.^8^, ^9^ Surgical drainage is indicated in cases of ruptured abscess, peritonitis, or an associated surgical cause. Liver abscesses, especially those caused by *Klebsiella pneumoniae*, have been associated with an increased risk of gastrointestinal malignancy.^10^ Colonoscopy is often recommended in these patients to assess for an underlying neoplasm.

Symptomatic treatment of persistent hiccups alone may result in delayed or missed diagnosis, which could be life‐threatening. This case highlights the importance of thoroughly investigating persistent or intractable hiccups for an underlying occult cause including the possibility of a PLA to prevent a delay in the diagnosis and treatment of this highly fatal disease.

### Informed Consent

Written informed consent has been obtained.
